# Expanding lignin thermal property space by fractionation and covalent modification†

**DOI:** 10.1039/d3gc01055d

**Published:** 2023-07-13

**Authors:** Luke A. Riddell, Floris J. P. A. Enthoven, Jean-Pierre B. Lindner, Florian Meirer, Pieter C. A. Bruijnincx

**Affiliations:** a Utrecht University, Organic Chemistry & Catalysis, Institute for Sustainable and Circular Chemistry, Faculty of Science Utrecht The Netherlands p.c.a.bruijnincx@uu.nl; b BASF SE, Group Research Carl-Bosch-Str. 38 67056 Ludwigshafen am Rhein Germany; c Utrecht University, Inorganic Chemistry & Catalysis, Debye Institute for Nanomaterial Science and Institute for Sustainable and Circular Chemistry, Faculty of Science Utrecht The Netherlands

## Abstract

To fully exploit kraft lignin's potential in material applications, we need to achieve tight control over those key physicochemical lignin parameters that ultimately determine, and serve as proxy for, the properties of lignin-derived materials. Here, we show that fractionation combined with systematic (incremental) modification provides a powerful strategy to expand and controllably tailor lignin property space. In particular, the glass transition temperature (*T*_g_) of a typical kraft lignin could be tuned over a remarkable and unprecedented 213 °C. Remarkably, for all fractions the *T*_g_ proved to be highly linearly correlated with the degree of derivatisation by allylation, offering such tight control over the *T*_g_ of the lignin and ultimately the ability to ‘dial-in’ this key property. Importantly, such control over this proxy parameter indeed translated well to lignin-based thiol–ene thermosetting films, whose *T*_g_s thus covered a range from 2–124 °C. This proof of concept suggests this approach to be a powerful and generalisable one, allowing a biorefinery or downstream operation to consciously and reliably tailor lignins to predictable specifications which fit their desired application.

## Abstract 2

To further unlock kraft lignin's potential in material applications, we show that tight control can be achieved over a key physicochemical lignin parameter, its *T*_*g*_, which largely determines, and serves as proxy for, the properties of lignin-derived thermoset materials.

## Graphical abstract

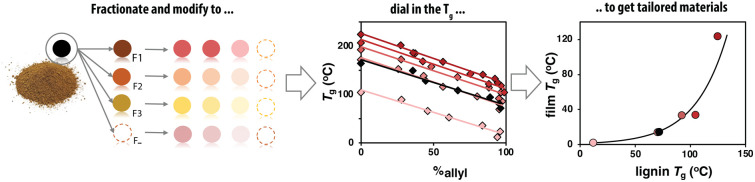

## Introduction

Efficient and viable biorefining necessitates the valorisation of lignocellulose's most complex and variable component, lignin. Current industrial delignification methods focus on accessing high purity carbohydrate streams to the detriment of the structure of the resulting technical, *i.e.* isolated, lignin.^1–5^ Of the delignification technologies, the kraft process is the most dominant, and structural analyses show kraft lignins to be a complex and heterogeneous combination of (a few remaining) common lignin inter-unit linkages, as well as kraft-specific structures and functionalities.^6–8^ Moreover, the distribution of functionalities and structures within the lignin is a function of molecular weight (MW). The complexity (and recalcitrance) of kraft lignin's polymeric structure is evident and suggests that kraft lignin valorisation *via* lignin-to-materials strategies, as opposed to lignin-to-chemicals *via* depolymerisation,^5,9^ perhaps is a more fruitful endeavour. Whilst important advances have been made in the many efforts focussing on direct lignin-to-materials approaches, which include lignin application in thermoplastics,^10,11^ road bitumen,^12,13^ films^14,15^ and polymer composites/blends,^16–18^ the pervasive problem of lignin's intrinsic functional group, linkage and MW heterogeneity has generally hampered many valorisation efforts. Indeed, intra-sample variation often leads to ill-defined and undesirable properties of the as-is technical lignin, as each individual component of the lignin contributes to the average with property values that span over a broad range, making it challenging to fit the lignin to an application. As an example, in lignin-polymer blends, this lack of property homogeneity (*e.g.* in solubility^19–21^) between the lignin (macro)molecules within a sample can lead to incompatibility between the lignin and the material matrix.^10,22^ In particular, kraft lignins are often not well suited for direct use in materials applications as they give rise to brittleness and themselves are only partially soluble in many conventional solvents, instead requiring less desirable solvents (*e.g.* DMF, DMSO) for total dissolution.^19^ Clearly, methods that (1) reduce lignin heterogeneity and (2) allow for fine-tuning the properties of a homogenised lignin sample would provide a powerful tool for more effective lignin valorisation. One often-used method to reduce heterogeneity is by fractionation.^23,24^ In one example, Duval *et al.* efficiently obtained lower-dispersity fractions from LignoBoost kraft lignin by sequential solvent extraction.^19^ Fine-tuning the properties of a (typically non-fractionated) lignin in turn is typically done by modification of the functional groups on the lignin. For example, Koivu *et al.* demonstrated the impact of fatty acid chain length and loading upon a lignin's glass transition temperature (*T*_g_).^25^ While both fractionation and modification are potentially powerful, the two methods are rarely combined in systematic fashion.

**Fig. 1:**
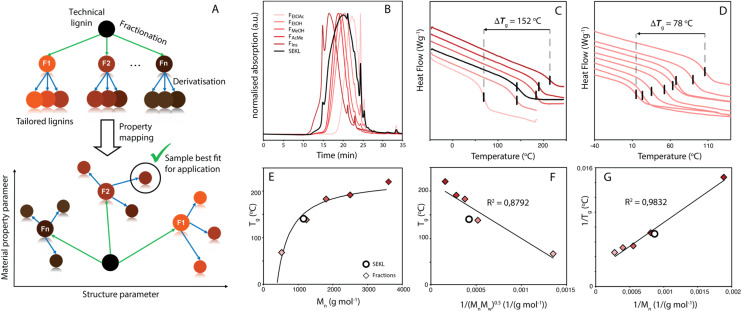
(a) Strategy for informed lignin selection by fractionation and derivatisation (b) normalised GPC traces of SEKL (black) and its subsequent fractions *F*_EtOAc_, *F*_EtOH_, *F*_MeOH_, *F*_AcMe_, *F*_Ins_ (red gradient) (c) DSC traces of SEKL (black) and its fractions (red gradient) (d) DSC traces of *F*_EtOAc_ and its allylated derivatives *F*_EtOAc_ − 0 to *F*_EtOAc_ − 6. (e) Flory-Fox fit for SEKL and fractions (f) Flory-Fox-Ogawa fit for SEKL and fractions (g) Fox-Loshaek fit for SEKL and fractions. Note that for (c) and (d), traces were shifted vertically for clarity.

The ability to ‘dial-in’ a property is particularly important, as the feedstock typically must meet product needs and not *vice versa*. Hence with a chosen performance parameter in mind, exploration of its property space is thus an important exercise in outlining the potential and flexibility of a feedstock material for application (Fig. 1a). Once the property space is mapped, correlating the relation between structural and material properties would provide better understanding and control over the latter, allowing for informed tailoring of the lignin to suit specific applications. In particular, the *T*_g_ is a key physicochemical characteristic as it determines the suitability of lignin use in many possible applications. As the *T*_g_ defines the temperature of a material's transition between glassy and brittle, to ductile and elastomeric, it can also be considered a proxy for other industrially relevant materials performance parameters (*e.g.*, viscosity). Homogenisation of the lignin *via* fractionation combined with partial/full modification of the lignin could considerably extend *T*_g_ space depending on the nature or degree of modification.

In addition to property homogenisation and adjustment, insertion of reactive handles is typically also required for downstream applications. For this, the –OH functionalities are most often targeted using a wide array of modification reactions, which have the added effect of altering the physicochemical properties of the lignin as well.^25–30^ Some recent examples have shown how either lignin fractionation, modification, or in rare cases fractionation combined with a single (full) modification can greatly improve applicability for materials use.^31–35^ Here, we build on this approach and present a powerful, translational strategy that systematically combines fractionation and incremental modification to explore, expand and fully map the physicochemical parameter space of a chosen lignin (Fig. 1a). Using the well-established Williamson-type phenol etherification to introduce reactive allyl handles for application in thiol–ene based thermosets, we demonstrate that by combining these two relatively simple approaches, lignin property space can be systematically and predictably expanded upon and in fact, controlled by the appropriate degree of modification of the proper fraction.^32,35^ This opens up avenues for lignin utilisation in material applications for which it hasn't previously been considered. Rewardingly, the control over the lignin's properties is directly reflected in its derived materials, *i.e.*, in the *T*_g_ of the thermosetting films. Such thermosets can be used in a wide range of applications, including in shape-memory materials,^36^ tissue scaffolds,^37^ coatings,^38^ and aerospace resins,^39,40^ thus providing many potential avenues for the valorisation of an underutilised bio-based feedstock.

## Results and discussion

### Fractionation, allylation and NMR analysis

The Lineo Classic lignin (SEKL) by Stora Enso is a commercially available softwood kraft lignin produced on an industrial scale and is thereby a relevant material for study. SEKL was fractionated by sequential extraction with ethyl acetate (EtOAc), ethanol (EtOH), methanol (MeOH) and finally acetone (AcMe), yielding four soluble fractions (*F*_EtOAc_, *F*_EtOH_, *F*_MeOH_, *F*_AcMe_) and a final insoluble residue (*F*_Ins_) of improved *Đ* (Fig. 1b, Table S3†). These were then fully structurally characterised, see ESI† for details. Note that this sequence of organic solvents has been shown to be effective for fractionation of un-modified softwood kraft lignin.^19,32,35^ Use of a solvent series may result in a relatively high *E*-factor, but this can be avoided by distillation recovery to regenerate the fractionating solvents, overall closing the solvent loop. Without solvent recovery, however, fractionation with a solvent series is less green than, *e.g.*, sequential precipitation methods,^41^ or ultrafiltration methodologies.^42^ Sequential precipitation methods, which have the disadvantage of generating contaminated water waste, have generally been explored for fractionation of modified lignins.^41^ Ultrafiltration methods are promising but suffer from filtrate flux decay due to membrane fouling, requiring its regeneration through cleaning. Overall, each fractionation method clearly has certain benefits and drawbacks which must be considered prior to selection of the method.

**Fig. 2:**
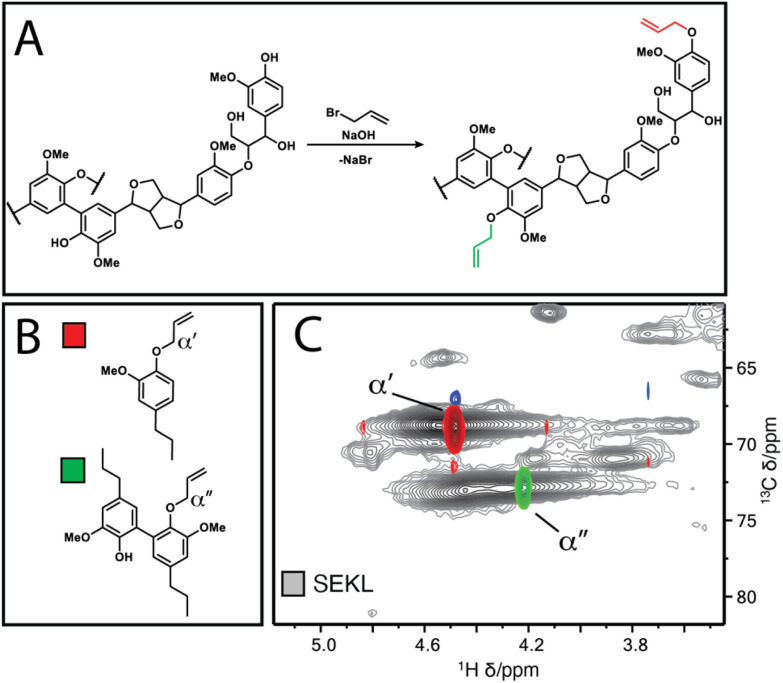
(a) Williamson allylation of lignin (b) Model compounds 1 & 2 (c) overlay of HSQC spectra of compounds 1 (red-blue) & 2 (green-orange) with SEKL (grey), respectively.

The fractions were then each allylated to different degrees (samples denoted as *F*_solvent_ − *X*, with higher *X* denoting higher degree of allylation). Post-allylation, the lignin samples were obtained as coloured powders, with a general brightening of the colour from brown to yellow with increasing %_allyl_; only *F*_EtOAc_−6 was obtained as a highly viscous liquid (Fig. S2†). The %_allyl_ is defined by the difference in the total phenolic hydroxyl group content prior/post-allylation, as the derivatisation method is almost entirely selective for phenolic OH allylation (Fig. 2a). The phenolic OH content was quantified as a function of allylation degree by ^31^P NMR after phosphitylation of the (remaining) –OH groups, taking into account the additional mass from the new pendant groups, as described elsewhere.^25^ HSQC NMR analysis confirmed successful selective allylation of the phenolic hydroxyl functionalities, with few other changes to the structure being observed. Notably, two distinct resonances differing in both ^1^H and ^13^C shift were observed for the allyl α(C–H) correlation, corresponding to G-units and 5-substituted G-units respectively, as identified by overlay of the HSQC spectra of allylated model compounds and of SEKL (Fig. 2c), confirming a previous suggestion that this arises due to condensation modes of the G-rings by Jawerth *et al.*^32^

### Thermal properties

Kraft lignins typically bestow rigidity and brittleness to materials, owing to their intricate hydrogen bonding network,^43,44^ and polyaromatic and highly condensed structure,^7,45^ resulting in high *T*_g_ values, much above room temperature. As anticipated, the *T*_g_ values obtained by modulated differential scanning calorimetry (MDSC) proved to be strongly dependent on both MW and %_allyl_. The *T*_g_ values of fractions and SEKL are well described by the Flory-Fox relationship^46^ (Fig. 1d), previously shown to be a remarkably good model for both organosolv and kraft lignins.^42,47,48^ Alternative relationships that take dispersity (*Đ*) (Flory-Fox-Ogawa,^49^Fig. 1f) and crosslinking (Fox-Loshaek,^50^Fig. 1g) into account have been applied to lignin as well;^47^ in particular the latter relationship gave an excellent fit for our sample set, lending further credence to the crosslinked nature of SEKL as a typical kraft lignin. Fractionation alone thus already extended *T*_g_ property space by 152 °C with *F*_EtOAc_ and *F*_Ins_ showing the lowest and highest measured *T*_g_s of 68 °C and 219 °C, respectively. The *T*_g_ of as-is SEKL is 141 °C, an intermediate value between the EtOH and MeOH fractions but given that SEKL has a lower *M*_n_ than *F*_EtOH_, the larger *T*_g_ of SEKL is due to its *Đ*.

**Fig. 3:**
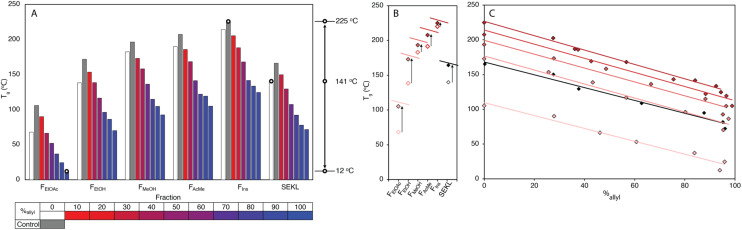
(a) Spread of *T*_g_ values for SEKL and its fractions through allylation, including control precipitation experiments for each fraction in grey (b) impact of control precipitation on SEKL and fractions (c) mapping of *T*_g_ values for SEKL and its fractions as a function of %_allyl_.

Systematic allylation allowed for further coverage and extension of property space. Notably, in addition to allowing application-tailored *T*_g_ values, the sample set also revealed strong structure–property relationships between %_allyl_ and *T*_g_. Overall, the lowest *T*_g_ value was found to be that of *F*_EtOAc_−6, the most highly derivatised *F*_EtOAc_ sample with *T*_g_ of 12 °C, a reduction of 56 °C when compared to the non-allylated EtOAc fraction (*F*_EtOAc_). The highest value was initially found to be from *F*_Ins_, yielding an expanded Δ*T*_g_ of 202 °C. The spread of values shown in Fig. 3a is quite remarkable when compared to literature values for fractionation (of an organosolv lignin) alone (∼150 °C),^48^ or for partial derivatisation of the same softwood kraft lignin with fatty acids (∼100 °C).^25^ Correlation plots of *T*_g_*vs.* %_allyl_ for each fraction's allyl derivatives, (Fig. 3c), show a remarkably strong linear relationship for all fraction series. The drop in *T*_g_ upon increasing %_allyl_ is considered a consequence of the loss in hydrogen bonding capacity of lignin, which has been identified as a key factor in determining the *T*_g_ of a lignin.^25,26,47^ While strongly linear relationships between the *T*_g_ and %_allyl_ were observed for all series, the non-derivatised samples (*e.g. F*_EtOAc_), as obtained from fractionation, did not (initially) fit the trends: all showed a lower *T*_g_ than anticipated from the fit through the derivatised samples. Inclusion of these samples would heavily skew the fit towards a curved, downward trajectory, similar to that reported by Duval and Avérous.^51^ The observed deviation from linearity could however be readily rationalised and attributed to incomplete precipitation of all lignin during recovery (*i.e.* a minor “fraction” of small components was not recovered). Control precipitation-recovery experiments conducted under reaction conditions without addition of allyl bromide yielded control samples (with suffix “−0”). These samples showed increased *T*_g_ values which fit excellently with the linear trends established with the derivatised samples (Fig. 3b & c; Table S5†). As expected, the difference with the control is larger for the lower *M*_w_ fractions, as with these samples, more small lignin fragments are present to begin with. Inclusion of the control samples further expanded the property space, with *F*_Ins_−0's *T*_g_ = 225 °C, yielding an overall Δ*T*_g_ = 213 °C. The maximum value afforded by this sample can indeed be considered as an “upper-limit” value to the property space of the Stora Enso lignin when treated under the fractionation and derivatisation conditions herein. Fig. 3c also shows a striking and unexpected co-linearity between each series, suggesting that the influence of incremental allylation appears to be uniform for each fraction, *i.e.* regardless of MW. This is tentatively attributed to the influence of aromatic-derived hydrogen bonding, a particularly dominant force in determining lignin's *T*_g_, but this requires further investigation. Regardless, the strong structure–property correlation signifies a level of control over the physical parameters of the lignin and hence predictability of properties when generating new samples.

**Fig. 4:**
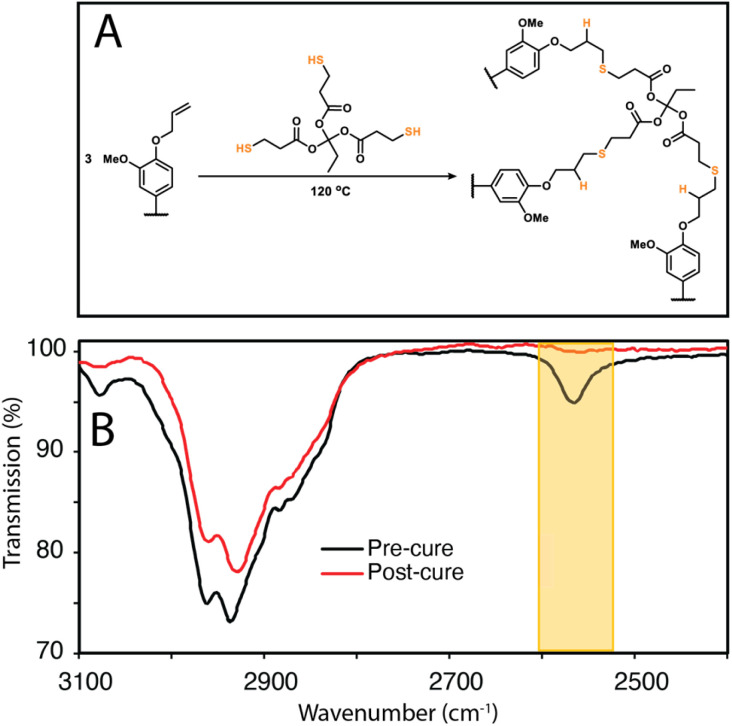
(a) Thiol–ene crosslinking reaction between lignin and TPTM (b) ATR-FTIR comparison of mixture of SEKL with TPTM before and after curing at 120 °C for 20 h.

**Fig. 5:**
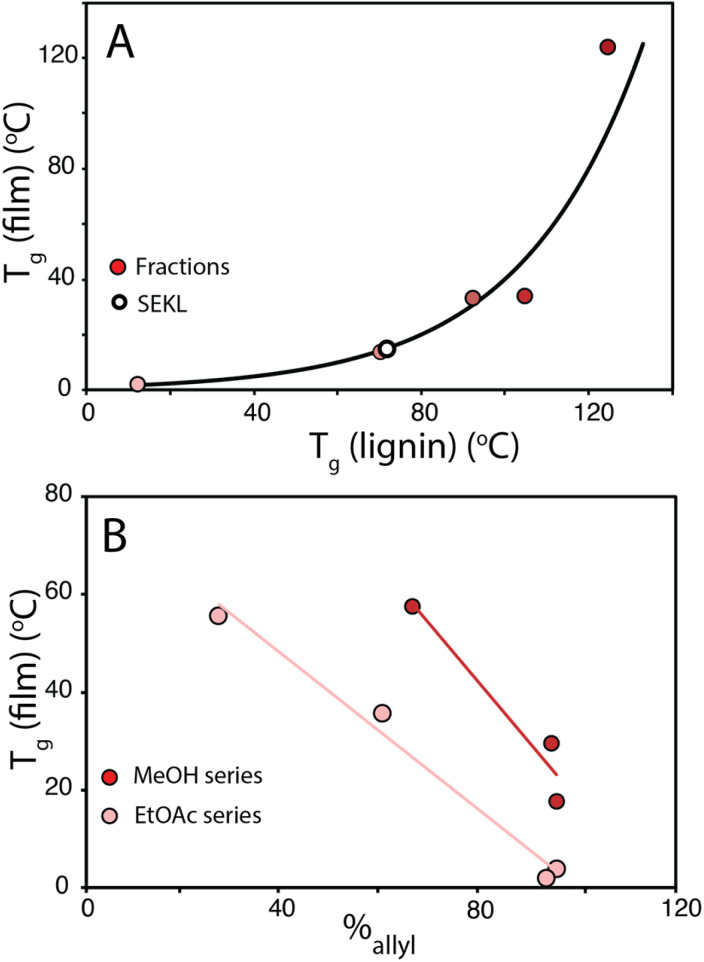
(a) *T*_g_ of films plotted as a function of their precursor sample (*F*_x_ − 6) *T*_g_ value (b) *T*_g_ of *F*_EtOAc_ and *F*_MeOH_ derived films as a function of %_allyl_.

To show that control over lignin *T*_g_ translates to application, a thiol–ene click reaction with was used to produce films with the *F*_X_ − 6 series of samples, according to the protocol of Lawoko and co-workers with trimethylolpropane tris(3-mercaptopropionate) (TPTM) as the crosslinking agent (Fig. 4).^32,33,35^ The resulting films were orange-brown in colour (Fig. S2†) and varied in their opacity and tactility, from the *F*_EtOAc_ − 6 film (*T*_g_ = 2 °C) which was a transparent and highly flexible solid, to the *F*_Ins_ − 6 film (*T*_g_ = 124 °C) which was transparent and somewhat brittle. The observed physical behaviours are reflected well in the *T*_g_ of the films and a range in Δ*T*_g_ of 122 °C was observed across the samples. There was also a difference in how effectively the modified lignins could disperse within the reaction mixture. In particular, the *F*_Ins_ − 6 was only partially soluble in EtOAc, leading to poor dispersion and aggregate formation within the resulting film, hence the poor optical features of this sample. Notably, the film's *T*_g_ was found to clearly depend on the precursor (modified) lignin *T*_g_ (Fig. 5a), showing that the fractionation methodology, and therefore the lignin MW serves well to modify the resulting film *T*_g_. Additionally, A blank reaction was performed on sample SEKL-1 under the above conditions, with the quantity of TPTM appropriate for sample SEKL-6; this material indeed showed poor solubility and did not form a film as expected, given lignin's relatively low abundance of unsaturated bonds (Fig. S4†). Finally, thiol–ene films were synthesised with selected samples from the *F*_EtOAc_ and *F*_MeOH_ series (see Table S6†). All but 1 lignin sample fully dissolved and formed homogeneous materials, with *F*_MeOH_ − 1 being the exception. Film formation was unsuccessful with this fraction and the material omitted from the series. Mapping the film *T*_g_ values of these thermosets as a function of their lignin's %_allyl_ (Fig. 5b) again showed a linear correlation, highlighting the strong influence of %_allyl_ on the resulting thermosetting films’ properties.

## Conclusions

The combined fractionation–modification approach demonstrates how well the properties of a lignin can be controlled. The strong correlation of *T*_g_ with *M*_n_ for each fraction showed that the lignins well fit the Flory-Fox and Fox-Loshaek models, with the latter providing an indication of crosslinking within the lignin structure. A total *T*_g_ property space of 213 °C across a range of 12–225 °C was achieved, considerably larger than seen before elsewhere in the literature for a single lignin. Furthermore, remarkably strong linear relationships between increasing %_allyl_ and decreasing *T*_g_ are reported for each of the fraction series, with notable collinearity between each series, suggesting that phenolic H-bonding is dominant in its influence over lignin *T*_g_. Finally, thiol–ene thermosetting resins were produced to validate the utilisation of a lignin's property as a proxy parameter for resultant materials’ properties. This concept was shown to be translated well to the example thermosets, with Δ*T*_g_ = 122 °C, covering a range from 2–124 °C. Much like with the lignins themselves, correlations were found between increasing %_allyl_ of the lignin sample with decreasing film *T*_g_, displaying excellent tuneability of the resulting material's properties. The systematic nature of the methodology and the strong structure–property relationships uncovered here reveal the possible levels of control over the lignin's properties. Fundamentally, this study (as a proof of concept) shows that within a property space range, lignins can be consciously tailored towards a desired material specification, thus providing a powerful toolbox to guide (industrial) end users towards fitting their lignin to a range of potential applications.

## Conflicts of interest

There are no conflicts to declare.

## Supplementary Material

GC-025-D3GC01055D-s001
