# Supplementary material for: Expanding lignin thermal property space by fractionation and covalent modification
Source: Green Chem. 2023 Jul 13;25(15):6051–6. doi: 10.1039/d3gc01055d (PMC10389295; doi:10.1039/d3gc01055d)
Supplement: GC-025-D3GC01055D-s001 [file GC-025-D3GC01055D-s001.pdf]

Electronic Supplementary Material (ESI) for Green Chemistry.
This journal is © The Royal Society of Chemistry 2023

Supporting Information

Expanding Lignin Thermal Property Space by Fractionation and Covalent Modification

Luke A. Riddell,[a] Floris J. P. A. Enthoven,[a] Dr. Jean-Pierre B. Lindner,[b] Dr. Florian Meirer,[c] Prof. dr. Pieter C. A. Bruijnincx,[a], *

[a] L.A. Riddell, F.J.P.A. Enthoven, Prof. dr. P.C.A. Bruijnincx
Utrecht University, Organic Chemistry & Catalysis, Institute for Sustainable and Circular Chemistry, Faculty of Science, Utrecht (The Netherlands)

[b] Dr. Jean-Pierre B. Lindner
BASF SE, Group Research
Carl-Bosch-Str. 38, 67056 Ludwigshafen am Rhein, Germany

[c] Dr. F. Meirer
Utrecht University, Inorganic Chemistry & Catalysis, Debye Institute for Nanomaterial Science and Institute for Sustainable and Circular Chemistry, Faculty of Science, Utrecht (The Netherlands)

Contents

Experimental 3
- Chemicals 3
- Sample Description 4
- Equipment 4
- Gel Permeation Chromatography (GPC) 4
- Modulated Differential Scanning Calorimetry (MDSC) 4
- Attenuated Total Reflectance Fourier Transform Infra-red Spectroscopy (ATR-FTIR) 4

Synthetic Methods 5
- Fractionation[3] 5
- Allylation of Lignin[4] 5
- Thiol-ene Cross-coupling of Lignin[5] 5
- Synthesis of 3,3'-dimethoxy-5,5'-dipropyl-[1,1'-biphenyl]-2,2'-diol[6] 5
- Allylation of model compounds to form (1) and (2) 5
- Model Compound Characteristics 6
  - 1-(prop-1-ene-3-oxy)-2-methoxy-4-propylphenol (1) 6
  - 2'-(prop-1-ene-3-oxy)-3,3'-dimethoxy-5,5'-dipropyl-[1,1'-biphenyl]-2-ol (2) 6

NMR Methods 6
- Quantitative ^{31}P NMR[8] 6
- Heteronuclear Single Quantum Coherence (HSQC) NMR 7

Fractionation, NMR & DSC Data 7
- NMR & MDSC Data 7

Lignin Samples 11

Thiol-ene Films 12

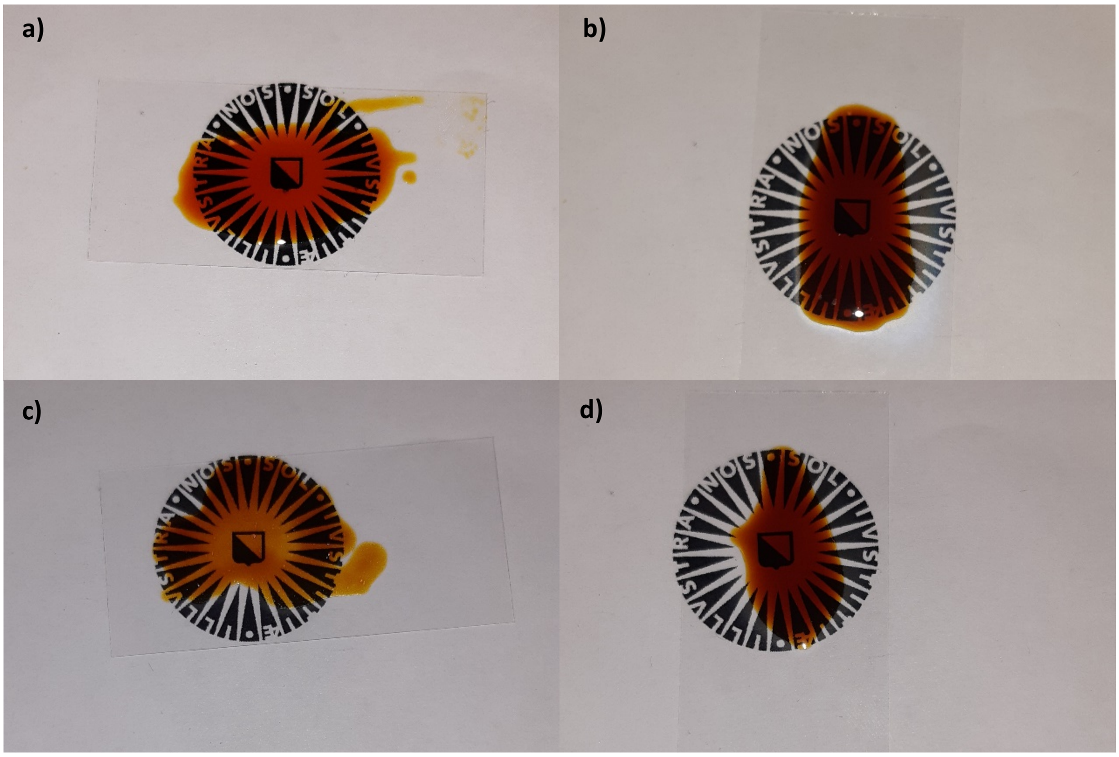

Figure S3. (a) F_{EtOAc}-6 derived thiol-ene film, (b) F_{MeOH}-6 derived thiol-ene film, (c) F_{Ins}-6 derived thiol-ene film, (d) SEKL-6 derived thiol-ene film

.... 12

References 13

Experimental

Chemicals

Softwood Stora Enso (LignoBoost) Kraft lignin was provided by BASF. The lignin was not subjected to any thermal or chemical pre-treatment before use in any procedure unless explicitly stated. Technical grade ethanol, methanol, ethyl acetate, acetone and toluene were obtained from VWR Chemicals. Sodium hydroxide (≥99%) was also obtained from VWR Chemicals. Pyridine (≥99%), and stabilised allyl bromide (99%) were obtained from Acros Organics. Deuterated chloroform (D, 99.8%) and deuterated pyridine (99.5%) were provided by Buchem. Cyclohexanol (99%) and 2-chloro-4,4,5,5-tetramethyl-1,3,2-dioxaphospholane (TMDP) (95%) were provided by Sigma Aldrich and chromium (III) acetylacetonate ($Cr(acac)_3$) (97%) was provided by Fisher Scientific. Trimethylolpropane tris(3-mercaptoprionate) (>95%) was obtained from Merck. Silica gel 60 (230-400 mesh) was also obtained from Merck.

Sample Description

For naming of samples, suffixes were appended to fraction names to distinguish between allylated samples, where non-allylated is denoted with 0, and sequential numbers indicate higher allyl bromide loading (1-6); e.g., the non-allylated methanol fraction sample is "F_{MeOH}-0", and the highest loading F_{MeOH} is "F_{MeOH}-6".

Equipment

All NMR measurements were recorded at 25 °C on either a Varian VNMRS 400 with a PFG probe, or an Agilent MRF400 spectrometer equipped with a OneNMR probe and Optima Tune system at 400 MHz. NMR samples were measured in standard 5 mm-OD NMR tubes and chemical shifts (δ) are reported in ppm. Acquired data was analysed and processed using Mestrenova (version 14.2.0-26256). DSC measurements were recorded on a TA instruments Discovery DSC and analysed within the TA instruments Trios software (v5.1.1.46572). GPC measurements were recorded on a Waters Alliance e2695 Separations Module equipped with 3 x PLGel mixed-E columns in series, a guard column and a Waters 2489 UV/visible Detector. ATR-FTIR was measured on a PerkinElmer UATR Two and processed using PerkinElmer Spectrum IR (version 10.6.2.1159).

Gel Permeation Chromatography (GPC)

Lignin samples were acylated prior to GPC measurements to ensure proper dissolution in the eluent. Lignin (50 mg) was dissolved in pyridine/acetic anhydride (1:1, 1 mL) and stirred overnight at room temperature. The acylated sample was recovered azeotropically, first by addition of toluene (500 µL x 5), then with EtOH (500 µL x 5) to yield a dry brown powder. Some samples required further addition of toluene (3 times) and EtOH (3 times) to fully remove trace impurities. The acylated samples were then dissolved in the tetrahydrofuran (THF) eluent spiked with acetic acid (1.5 mg/mL, 0.1% AcOH/THF v/v) and filtered with a 45 µm PTFE syringe filter before GPC analysis. UV detection was performed at a wavelength of 280 nm and molecular weight determinations were based on calibrations with polystyrene standards (M_n = 104, 208, 312, 416, 520, 625, 729, 833, 937, 1930, 2900, 3790, 6180, 10110, 16500, 24600, 38640 $gmol^{-1}$). As polystyrene evidently doesn't have the same structure as lignin, its use as proxy calibration standard means that reported molecular weights need to be interpreted with some caution in relation to a sample's absolute molecular weight. The use of PS is nevertheless common and allows for benchmarking with existing literature on kraft lignins and their derivative fractions.[1,2]

Modulated Differential Scanning Calorimetry (MDSC)

Lignin (5 mg) was weighed into a Tzero low-mass aluminium pan, fitted with a Tzero lid which was drilled to produce a venting hole (0.8 mm). Once the pan was loaded into the DSC cell, the sample was equilibrated to 20 °C. Two heating cycles were then performed. For the first cycle, the sample was heated to 105°C at 10 °C/min, was held isothermal for 20 min, cooled to -50 °C at 10 °C/min and held isothermal for 1 min to anneal the sample, remove any residual solvents and to erase the sample's thermal history. Data acquisition was performed on the second heating cycle, whereby the temperature was ramped at 3 °C/min from -50 °C to 190/250 °C with a sinusoidal temperature modulation of 0.66 °C per 50 s. Each sample was measured in duplicate, and the average value was taken.

Attenuated Total Reflectance Fourier Transform Infra-red Spectroscopy (ATR-FTIR)

Before each measurement, a background measurement was taken to remove residual unnecessary signals. A small quantity of sample was placed onto the diamond aperture, ensuring total coverage. The toner arm was lowered to press the sample onto the diamond and the spectrum was measured with a spectral range of

600-4000 cm^{-1} with 4 co-added scans. The resulting spectra were then background corrected within the PerkinElmer Spectrum IR software.

Synthetic Methods

Fractionation[3]

100 g of Stora Enso Kraft lignin, ground by pestle and mortar and sieved (450 μm), was suspended in 1000 mL of ethyl acetate in a round bottom flask and stirred for 2 h at room temperature. The insoluble fraction was then separated from the solution by vacuum filtration. The insoluble residue was then air-dried, re-suspended, stirred and extracted under the same conditions with 1000 mL of the subsequent solvent. The soluble fractions were isolated by removal of solvent *in vacuo*. To minimise residual traces of organic solvent, each fraction was wetted with water to form a paste and dried through rotary evaporation, followed by drying under a gentle stream of compressed air overnight, followed by 24 h in a vacuum oven at 65 °C. The chosen solvent order was ethyl acetate, ethanol, methanol, and acetone, yielding four soluble fractions and an insoluble residual fraction.

Allylation of Lignin[4]

Lignin samples were hand ground with a pestle and mortar until a visually homogeneous particle size was achieved. The ground lignin (1 g) was dissolved in an acetone/NaOH solution (1:3, 40 mL). Different volumes of allyl bromide (0.11−3.29 equiv. vs total lignin [OH]) were carefully added through a septum, to the magnetically stirring mixture. The reaction was held at 40 °C for 5 h under a nitrogen atmosphere. The reaction mixture was then reduced *in vacuo* to remove the acetone and the crude mixture was acidified to pH 2 by adding 35% HCl dropwise. The resulting slurry was purified by centrifuging at 8000 rpm for 5 minutes, decanting the supernatant, followed by washing with demineralised water (10 mL), and centrifuging again. The washing steps were repeated two more times and afterwards the sample was air-dried under a gentle stream of compressed air overnight, followed by 24 h in a vacuum oven at 60 °C.

Thiol-ene Cross-coupling of Lignin[5]

Trimethylolpropane tris(3-mercaptoprionate) (0.333 equiv. vs allyl content of lignin) was added to approximately 60 mg of allylated lignin samples in a vial containing 135 μL EtOAc and stirred until homogenous. The mixture was then transferred via pipette to a microscopy glass viewing plate and the solvent left to evaporate for 30 minutes, before curing at 120 °C for 24 h.

Synthesis of 3,3'-dimethoxy-5,5'-dipropyl-[1,1'-biphenyl]-2,2'-diol[6]

An aqueous sodium acetate solution was prepared (18.0 g, 100 mL), after which 4-propylguaiacol (16.8 g, 0.10 mol) was added at room temperature. To this vigorously stirring suspension, an aqueous solution of $K_3[Fe(CN)_6]$ (35.0 g, 0.11 mol, 300 mL) was added dropwise. After total addition, the reaction was left to stir for 24 h, after which the mixture was extracted with DCM (4 x 200 mL). The organic layers were combined, dried with magnesium sulphate, filtered, and concentrated in vacuo to yield a white crystalline solid, which showed resonances in ^{13}C & ^{1}H NMR which corresponded to shifts, multiplicities, and peak integrations reported in the literature.[6]

Allylation of model compounds to form (1) and (2)

Modified from a generalised protocol.[7] To a stirred solution of phenolic compound (605 μmol) in acetone (8 mL) was added K_2CO_3 (0.166 g, 1.210 mmol), followed by allyl bromide (1.45 mmol, 125 μL) at room temperature. Stirring was then continued for 2 h under reflux, after which the reaction mixture was left to cool. The suspension was then filtered over a silica plug and reduced in vacuo. Following this, the solution was redissolved in DCM (8 mL) and extracted with H_2O (3 x 8 mL). Finally the conversion was checked by thin

layer chromatography (TLC) (hexane:EtOAc, 9:1, rf = 0.29) and if necessary, the product was purified by flash chromatography (hexane:EtOAc, 9:1).

Model Compound Characteristics

1-(prop-1-ene-3-oxy)-2-methoxy-4-propylphenol (1)

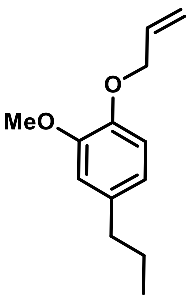

^{1}H-NMR (d_6-DMSO, 400 MHz): δ 0.85 (t, *J* = 7.3 Hz, 3H), δ 1.53 (m, *J* = 7.5 Hz, 2H), δ 2.46 (t, *J* = 7.6 Hz, 2H), δ 3.72 (s, 3H), δ 4.46 (dt, *J* = 5.4, 1.6 Hz, 2H), δ 5.20 (dq, *J* = 10.5, 1.5 Hz, 1H), δ 5.34 (dq, *J* = 17.3, 1.7 Hz, 1H), δ 6.00 (ddt, *J* = 17.3, 10.5, 5.3 Hz, 1H), δ 6.63 (dd, *J* = 8.1, 2.0 Hz, 1H), δ 6.76 (d, *J* = 2.0 Hz, 1H), δ 6.81 (d, *J* = 8.1 Hz, 1H). ^{13}C-NMR (d_6-DMSO, 101 MHz): δ 13.6 (CH_3), δ 24.2 (CH_2), δ 36.9 (CH_2), δ 55.5 (CH_3), δ 69.1 (CH_2), δ 112.5 (CH), δ 113.7 (CH), δ 117.2 (CH_2), δ 120.0 (CH), δ 134.1 (CH), δ 135.1 (CH), δ 145.7 (CH), δ 149.0 (CH).

2'-(prop-1-ene-3-oxy)-3,3'-dimethoxy-5,5'-dipropyl-[1,1'-biphenyl]-2-ol (2)

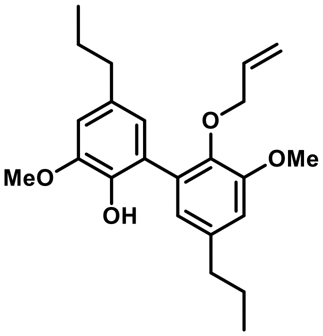

^{1}H-NMR (d_6-DMSO, 400 MHz): δ 0.90 (m, *J* = 7.3 Hz, 6H), δ 1.58 (m, *J* = 7.4 Hz, 4H), δ 2.48 (t, *J* = 7.6 Hz, 4H*), δ 3.80 (s, 6H), δ 4.22 (dt, *J* = 5.3 Hz, 2H), δ 4.96 (dq, *J* = 10.5, 1.9 Hz, 1H), δ 5.06 (dq, *J* = 17.2, 1.8 Hz, 1H), δ 5.70 (ddt, *J* = 16.3, 10.7, 5.5 Hz, 1H), δ 6.49 (d, *J* = 2.0 Hz, 1H), δ 6.56 (d, *J* = 2.0 Hz, 1H), δ 6.74 (d, *J* = 2.0 Hz, 1H), δ 6.81 (d, *J* = 2.1 Hz, 1H). ^{13}C-NMR (d_6-DMSO, 101 MHz): δ 13.6 (CH_3), δ 13.8 (CH_3), δ 24.1 (CH_2), δ 24.3 (CH_2), δ 36.9 (CH_2), δ 37.1 (CH_2), δ 55.7 (CH_3), δ 55.8 (CH_3), δ 73.0 (CH_2), δ 110.9 (CH), δ 111.7 (CH), δ 116.2 (CH_2), δ 122.6 (CH), δ 122.7 (CH), δ 125.4 (C), δ 131.7 (C), δ 132.6 (C), δ 134.8 (CH), δ 134.9 (CH), δ 137.0 (C), δ 141.6 (C), δ 143.3 (C), δ 147.2 (C), δ 141.6 (C), 152.2 (C).

* Peak overlaps with d_6-DMSO peak, ^{1}H NMR was re-run in $CDCl_3$ for integration of this resonance.

NMR Methods

Quantitative ^{31}P NMR[8]

The peak assignments and integration ranges were based on the work of Argyropoulos[8] and measurements were performed with the internal standard cyclohexanol, as reported previously by our group.[1,9,10]

A dry solvent mixture of deuterated chloroform/pyridine (1:1.6, v/v) was prepared and stored over 3 Å molecular sieves. Separate stock solutions of the internal standard (cyclohexanol, 19 mg/mL) and the paramagnetic relaxation agent (chromium (III) acetylacetonate, 5 mg/mL) were prepared using the solvent mixture. 500 µL of the solvent mixture was added to approximately 40 mg of accurately weighed lignin, followed by internal standard and relaxation agent solutions (200 µL & 50 µL, respectively) at room temperature. The sample was stirred for at least an hour and up to overnight to achieve total dissolution. Immediately prior to measurement, the sample was phosphitylated by addition of TMDP (100 µL) and the mixture was allowed to stir for 1 minute before transfer to an NMR tube. A standard phosphorous pulse program with a pulse angle of 45°, relaxation delay of 10 s and 512 co-added scans was used. Peak shifts were referenced by the sharp peak arising from reaction of residual water with TMDP at 132.2 ppm. Spectra were processed by application of a 3rd order polynomial baseline correction, followed by automatic phase adjustment, which provided adequate internal standard peak shape.

Heteronuclear Single Quantum Coherence (HSQC) NMR

Lignin (200 mg) was dissolved in 750 µL d_6-DMSO overnight. Spectra were recorded using a "gc2hsqcse" pulse sequence and optimised for 145 Hz ^1J with multiplicity editing turned off. The spectral width was -2 to 14 ppm in f2 (^{1}H) and -10 to 190 in f1 (^{13}C) with 256 scans in f2 and 64 scans in f1. For all spectra, a 3rd order polynomial baseline correction in both dimensions and automatic phase correction was performed. The spectra were apodised in f2 (LB = 0.3 Hz, GB = 0.1) and a sine squared function set to 90° was applied in both f1 and f2. Spectral shifts were referenced to the shift of the solvent peak for HSQC measurements. Peak assignments were based on existing literature.[9]

Fractionation, NMR & DSC Data

NMR & MDSC Data

Table S1. ^{31}P and MDSC data of SEKL, its fractions and their allylated derivatives; [a]mmol/g

Sample Name	Aliphatic OH[a]	5-Substituted ArOH[a]	Guaiacyl OH[a]	*p*-Hydroxyphenyl OH[a]	COOH[a]	Total PhOH[a]	Total OH[a]	$\%_{allyl}$	T_g (°C)	σT_g (°C)
F_{EtOAc}	1.4	2.3	3.9	0.3	0.7	6.5	8.5	0	68	0.420
F_{EtOAc}-1	1.1	1.6	2.7	0.2	0.8	4.5	6.4	28	90	0.015
F_{EtOAc}-2	0.8	1.1	2.0	0.1	0.7	3.2	4.7	46	66	2.92
F_{EtOAc}-3	0.9	0.9	1.2	0.1	0.6	2.2	3.6	61	53	0.900
F_{EtOAc}-4	0.9	0.5	0.3	0.0	0.6	0.8	2.4	84	37	1.61
F_{EtOAc}-5	1.1	0.2	0.0	0.0	0.5	0.2	1.8	96	24	1.15

F_{EtOAc}-6	1.0	0.2	0.1	0.0	0.3	0.3	1.6	94	12	2.60
F_{EtOH}	2.0	2.0	2.2	0.2	0.5	4.4	6.9	0	138	2.89
F_{EtOH}-1	1.5	1.4	1.6	0.1	0.5	3.2	5.1	26	154	0.215
F_{EtOH}-2	1.3	1.1	1.2	0.1	0.4	2.3	4.1	43	139	1.79
F_{EtOH}-3	1.5	0.9	0.7	0.1	0.4	1.7	3.6	57	117	0.400
F_{EtOH}-4	2.0	0.5	0.2	0.0	0.6	0.7	3.4	80	96	1.26
F_{EtOH}-5	1.5	0.1	0.0	0.0	0.4	0.1	2.4	97	86	2.35
F_{EtOH}-6	1.6	0.2	0.0	0.0	0.2	0.2	1.9	95	70	0.595
F_{MeOH}	3.2	2.0	2.8	0.2	0.4	4.9	8.4	0	183	0.580
F_{MeOH}-1	2.1	1.7	1.6	0.2	0.5	3.4	6.0	28	173	0.310
F_{MeOH}-2	1.8	1.2	1.1	0.1	0.4	2.3	4.5	49	158	1.02
F_{MeOH}-3	1.7	0.8	0.6	0.1	0.4	1.4	3.5	67	136	0.100
F_{MeOH}-4	2.0	0.3	0.1	0.0	0.4	0.5	2.9	88	115	0.615
F_{MeOH}-5	1.9	0.1	0.0	0.0	0.4	0.1	2.4	96	105	1.43
F_{MeOH}-6	1.8	0.2	0.0	0.0	0.2	0.2	2.2	95	93	0.585
F_{AcMe}	2.0	2.1	2.7	0.2	0.3	5.0	7.3	0	191	1.52
F_{AcMe}-1	1.6	1.5	1.4	0.1	0.3	3.0	5.0	37	186	0.0750
F_{AcMe}-2	1.7	1.4	1.1	0.1	0.4	2.7	4.8	43	169	0.750
F_{AcMe}-3	1.6	0.7	0.4	0.0	0.3	1.1	3.0	75	143	1.600
F_{AcMe}-4	1.8	0.4	0.1	0.0	0.4	0.5	2.7	88	122	0.750
F_{AcMe}-5	1.7	0.1	0.0	0.0	0.3	0.1	2.2	97	119	1.12
F_{AcMe}-6	1.5	0.0	0.0	0.0	0.0	0.0	1.6	99	105	0.100
F_{Ins}	2.7	1.6	1.3	0.2	0.3	3.0	6.0	0	220	5.47
F_{Ins}-1	2.1	1.1	0.9	0.1	0.3	2.1	4.5	27	203	2.86
F_{Ins}-2	2.4	1.0	0.7	0.1	0.3	1.8	4.5	36	187	1.55
F_{Ins}-3	2.1	0.7	0.4	0.1	0.2	1.2	3.6	57	168	7.50
F_{Ins}-4	1.8	0.3	0.1	0.0	0.4	0.4	2.7	84	142	2.21
F_{Ins}-5	2.5	0.2	0.0	0.0	0.3	0.2	3.0	92	134	0.740
F_{Ins}-6	2.5	0.1	0.0	0.0	0.1	0.1	3.0	94	125	1.45
SEKL	2.5	2.2	2.3	0.3	0.6	4.8	7.8	0	141	0.280
SEKL-1	1.7	1.3	1.5	0.2	0.4	3.0	5.5	27	150	0.660
SEKL-2	1.8	1.2	1.2	0.1	0.5	2.5	4.7	37	129	0.010
SEKL-3	1.7	0.7	0.6	0.1	0.4	1.4	3.5	63	109	1.300
SEKL-4	1.9	0.3	0.1	0.0	0.4	0.4	2.8	88	95	2.42
SEKL-5	1.8	0.1	0.0	0.0	0.4	0.2	2.4	95	82	3.98
SEKL-6	1.8	0.1	0.0	0.0	0.2	0.1	2.2	96	72	0.830

Table S6. Lignin samples used to form Thiol-ene thermosetting films

Sample	Film T_g (°C)
SEKL-6	15
F_{EtOAc}-1	56
F_{EtOAc}-3	36
F_{EtOAc}-5	4
F_{EtOAc}-6	2
F_{EtOH}-6	14
F_{MeOH}-3	58
F_{MeOH}-5	18
F_{MeOH}-6	[illegible]
F_{AcMe}-6	34
F_{Ins}-6	124

Table S2. HSQC NMR data of SEKL and its fractions, where S,G,H are given as a ratio. All units reported in /100Ar, using G_2 + $S_{2,6}$ integrals as internal standard

Lignin	S	G	H	SB1	SB5	β-5	β-O-4	β-β	β-β'	$X_γ$	$SR_β$	$DHCA_β$
F_{EtOAc}	4.0	91	4.6	3.6	8.4	0.4	1.1	0.9	0.4	1.5	1.6	3.7
F_{EtOH}	1.9	95	3.1	1.4	7.0	2.0	5.2	1.9	0.7	2.6	2.4	5.2
F_{MeOH}	1.6	95	3.8	0.8	7.0	2.6	6.4	2.4	0.8	2.7	2.1	4.9
F_{AcMe}	4.2	93	2.6	0.3	6.3	2.4	5.5	3.3	0.8	3.2	1.9	3.7
F_{Ins}	5.2	93	1.3	0.0	3.5	2.3	5.5	2.7	0.1	3.8	1.8	4.9
SEKL	3.6	93	3.4	2.0	8.4	1.3	3.5	1.6	0.6	2.7	2.0	4.0

Table S5. MDSC data of precipitated samples run under allylation reaction conditions without addition of allyl bromide

Sample Name	Control T_g (°C)	$σT_g$ (°C)
F_{EtOAc}-0	105	0.785
F_{EtOH}-0	173	0.965
F_{MeOH}-0	193	3.37
F_{AcMe}-0	208	0.230
F_{Ins}-0	225	1.76
SEKL-0	165	0.960

Table S3. Molecular weight data of SEKL and fractions as obtained by GPC

Sample	M_n (g mol-1)	M_w (g mol-1)	Đ
F_{EtOAc}	530	1033	1.95
F_{EtOH}	1237	2985	2.41
F_{MeOH}	1808	3899	2.16
F_{AcMe}	2498	5045	2.02
F_{Ins}	3609	10896	3.02
SEKL	1160	4841	4.17

Table S4. Yield comparisons from fractionation within this body of work and by [a] Giummerella *et al.*[11] and [b] Duval *et. al.*[3]

	Yield (%)		
Fraction	[a]	[b]	This work
SEKL	-	-	-
F_{EtOAc}	18	28	21
F_{EtOH}	24	20	27
F_{MeOH}	7	13	9
F_{AcMe}	20	6	6
F_{Ins}	31	27	34

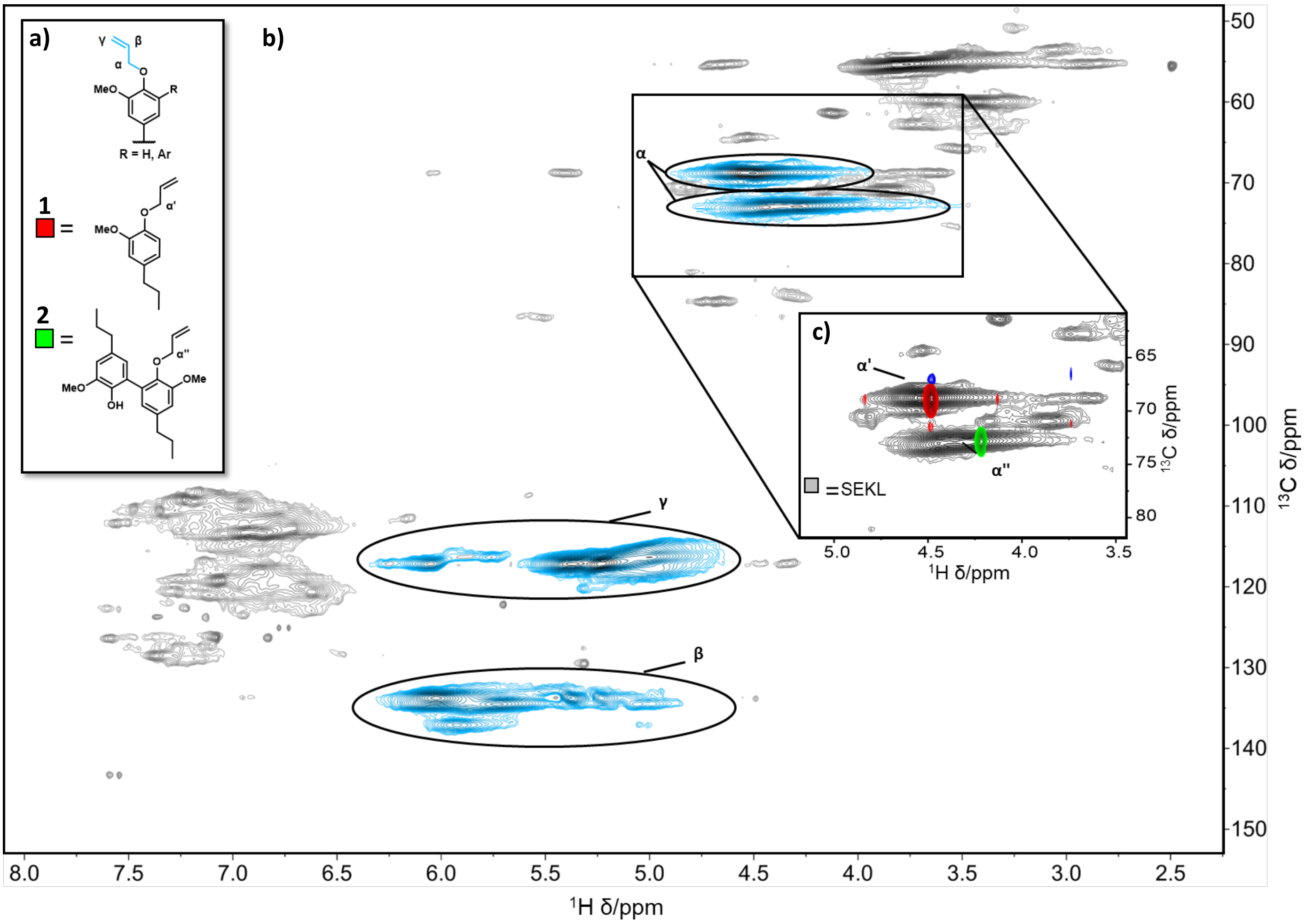

Figure S1. a) Allylated lignin fragment and allylated model compounds 1 (red) and 2 (green) b) Truncated HSQC NMR spectrum of SEKL-6 with the allyl C-H peaks highlighted in blue c) Zoom of the C-H$_{\alpha}$ correlation peaks of lignin overlaid with the peaks from model compounds 1 (red) and 2 (green)

Lignin Samples

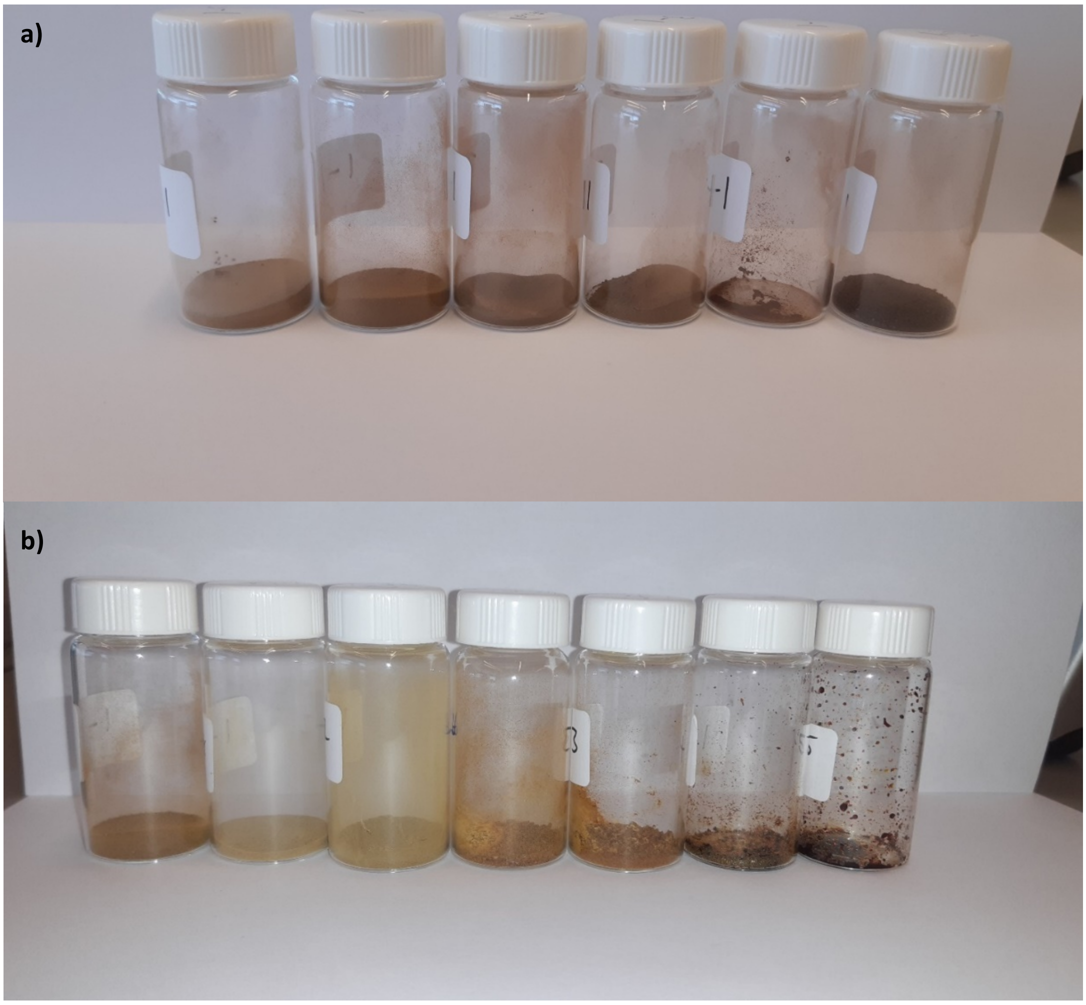

Figure S2. (a) Left to right: F_{EtOAc}, F_{EtOH}, F_{MeOH}, F_{AcMe}, F_{Ins} (b) Left to right: F_{EtOAc}, F_{EtOAc}-1, F_{EtOAc}-2, F_{EtOAc}-3, F_{EtOAc}-4, F_{EtOAc}-5, F_{EtOAc}-6

Thiol-ene Films

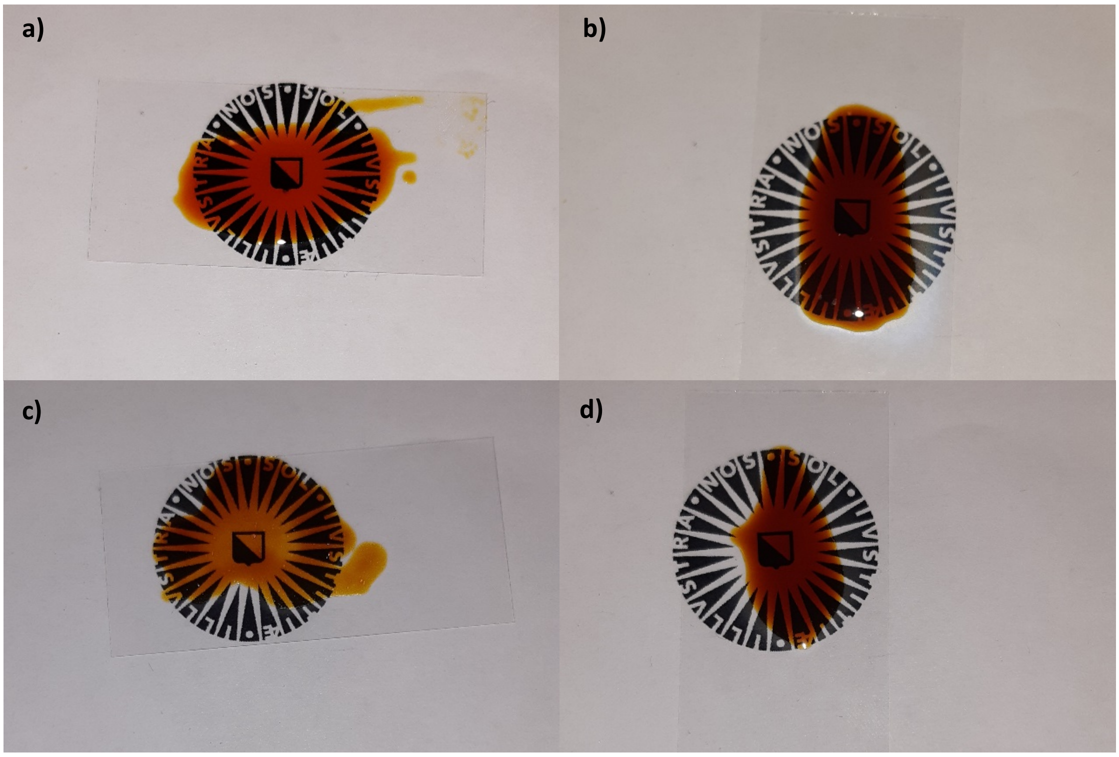

Figure S3. (a) F_{EtOAc}-6 derived thiol-ene film, (b) F_{MeOH}-6 derived thiol-ene film, (c) F_{Ins}-6 derived thiol-ene film, (d) SEKL-6 derived thiol-ene film

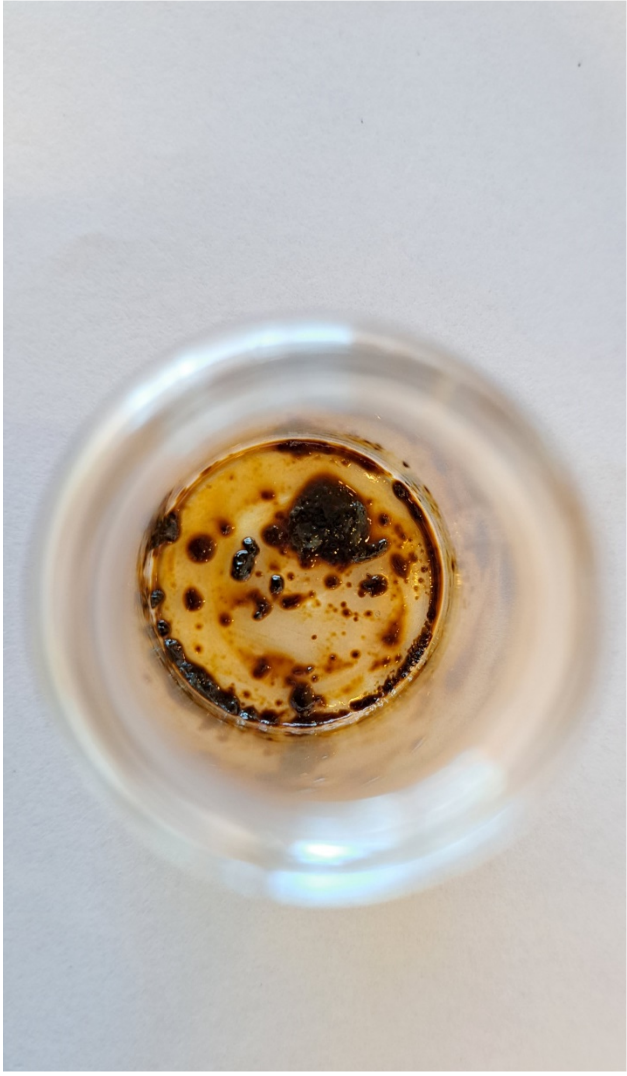

Figure S4. Blank reaction of SEKL-1 under the thiol-ene crosslinking reaction conditions

References

[1] C. S. Lancefield, S. Constant, P. de Peinder, P. C. A. Bruijnincx, *ChemSusChem* **2019**, *12*, 1139–1146.

[2] N. Giummarella, I. V. Pylypchuk, O. Sevastyanova, M. Lawoko, *ACS Sustain. Chem. Eng.* **2020**, *8*, 10983–10994.

[3] A. Duval, F. Vilaplana, C. Crestini, M. Lawoko, *Holzforschung* **2016**, *70*, 11–20.

[4] L. Zoia, A. Salanti, P. Frigerio, M. Orlandi, *BioResources* **2014**, *9*, 6540–6561.

[5] M. Jawerth, M. Johansson, S. Lundmark, C. Gioia, M. Lawoko, *ACS Sustain. Chem. Eng.* **2017**, *5*, 10918–10925.

[6] E. Subbotina, M. V. Galkin, J. S. M. Samec, *ACS Sustain. Chem. Eng.* **2017**, *5*, 3726–3731.

[7] M. Yoshida, M. Higuchi, K. Shishido, *Org. Lett.* **2009**, *11*, 4752–4755.

[8] D. S. Argyropoulos, *J. Wood Chem. Technol.* **1994**, *14*, 45–63.

[9] C. S. Lancefield, H. J. Wienk, R. Boelens, B. M. Weckhuysen, P. C. A. Bruijnincx, *Chem. Sci.* **2018**, *9*, 6348–6360.

[10] S. Constant, H. L. J. Wienk, A. E. Frissen, P. De Peinder, R. Boelens, D. S. Van Es, R. J. H. Grisel, B. M. Weckhuysen, W. J. J. Huijgen, R. J. A. Gosselink, P. C. A. Bruijnincx, *Green Chem.* **2016**, *18*, 2651–2665.

[11] N. Giummarella, P. A. Lindén, D. Areskogh, M. Lawoko, *ACS Sustain. Chem. Eng.* **2020**, *8*, 1112–1120.
